# Comparative Proteomics of Human Milk From Eight Cities in China During Six Months of Lactation in the Chinese Human Milk Project Study

**DOI:** 10.3389/fnut.2021.682429

**Published:** 2021-08-12

**Authors:** Ratna Nurmalita Sari, Jiancun Pan, Wenyuan Zhang, Yuanyuan Li, Huiquan Zhu, Xiaoyang Pang, Shuwen Zhang, Shilong Jiang, Jing Lu, Jiaping Lv

**Affiliations:** ^1^Institute of Food Science and Technology, Chinese Academy of Agricultural Science, Beijing, China; ^2^Nutrition and Metabolism Research Division, Innovation Center, Heilongjiang Feihe Dairy Co., Ltd., Beijing, China; ^3^PKUHSC-China Feihe Joint Research Institute of Nutrition and Healthy Lifespan Development, Beijing, China; ^4^School of Food and Health, Beijing Business and Technology University, Beijing, China

**Keywords:** Chinese human milk, human milk, cities, lactation period, proteomics

## Abstract

Human milk (HM) is the golden standard of infant nutrition that can protect immature body function and enhance nutrition metabolism to ensure infant growth. Region specificity and lactation period could change the protein composition in HM. In this research, proteomics analysis was used to compare proteomes across eight cities, namely Harbin, Lanzhou, Guangzhou, Chengdu, Jinhua, Weihai, Zhengzhou, and Beijing, which represented the northeast, northwest, southeast, southwest, east, and north and central regions of China,. Proteins varied significantly among the cities. These different proteins were mainly involved in the process of platelet degranulation, innate immune response, and triglyceride metabolic process, which might be due to different living environments. These differences also lead to variation in protection and fat metabolism from mothers to infants in different cities. Four proteins were expressed differently during 6 months of lactation, namely Dipeptidyl peptidase 1, Lysozyme C, Carbonic anhydrase 6, and Chordin-like protein 2. The changes in these proteins might be because of the change of growth needs of the infants. The findings from our results might help to improve the understanding of HM as well as to design infant formula.

## Introduction

Human milk (HM) is a complex liquid that contains variative compositions among mothers. It consists of true solutions, colloids, membranes, membrane-bound globules, and cells (1). The exclusive breastfeeding period of the first 6 months then continued up to 2 years is considered as the ideal standard for infant feeding (2). During the exclusive breastfeeding period, HM acts as the single source of nutrients that supports the immature immunity and metabolism of the infant (3). Meanwhile, the composition of HM is altered by many factors such as maternal factors, lactation stages, environmental exposures, regions, ethnicities, handling, and storage (1, 4).

Human milk contains myriad proteins that play a role in the biological process of infants such as immunological, antimicrobial, and developmental functions (5, 6). Recently, proteomics has become a robust approach to explore the overall biological function of HM proteins (7). Previously, proteomes variation of HM serum and milk fat globule membrane of individual mothers and variation of HM and ruminant's milk have been observed (3, 8–10). Those prior studies found that HM proteome contributed to various functions including immune protection, biological growth, and maturation of the digestive tract. HM profile, including lipid, oligosaccharides, and distinct patterns of microbiota varied across the population (11, 12). However, not so many studies emphasized the variation of protein composition, especially low abundant ones in the different regions. The variation of quantitative proteomes between Chinese and Dutch HM serum and the different proteomes across Chinese ethnicity and geographic location has been revealed (13, 14). They studied milk serum proteome of four different regions in China, mainly focused on the western region of China, namely Yunnan, Gansu, Xinjiang, and Inner Mongolia. To our knowledge, the milk proteins, especially low abundant proteins were not well-investigated in the eastern region of China.

Thus, HM proteomes in eight cities of China, which represented northeast, northwest, southeast, southwest, east, north, and central China, during the first 6 months of lactation were investigated in the present study by using proteomics methods with a bigger number of samples than prior studies. The result of this research will build in-depth understanding of proteomes in each city for further use, for instance, the development of infant formula for the infants that could not access sufficient HM.

## Materials and Methods

### Materials

All the reagents were provided in analytical grade and suitable for liquid chromatography–mass spectrometry (LC-MS/MS). The Bicinchoninic acid assay (BCA) kit was purchased from Biodee, China. Ammonium bicarbonate, dithiothreitol (DTT), and iodoacetamide (IAA) were obtained from Sigma, USA. Acetonitrile was obtained from Thermo Fisher, USA. Spectrometry grade of trypsin was purchased from Promega, USA.

### Sample Collection

The Chinese Human Milk Project (the CHMP study) recruited 1,800 participants from eight cities of Mainland China (Beijing, Guangzhou, Chengdu, Weihai, Lanzhou, Jinhua, Zhengzhou, and Harbin) to evaluate HM composition in the Chinese population. This present study used samples during 1–6 months of lactation and had been clinically registered in ClinicalTrials.gov with registration identifier NCT03675204.

The present study used cross-sectional sample by using one sample from one individual analyzed for one time. HM was collected at 9:00–11:00 in the morning from 15 to 180 days after delivery. The samples were collected from both the right and left sides of fully pumped breasts. The minimum size for each sample was 60 ml. The inclusion criteria were lactating mothers 25–35 years old, breast-fed infants 15–180 days old, physically healthy, non-smoking and non-alcoholic consumers, given birth to physically healthy infants, and signed informed consent forms. The detailed number of the samples is figured in Table 1. The obtained samples were frozen and transported to the lab and stored at a temperature of −80°C until further analysis.

**Table 1:** Number of samples obtained from the hospitals in eight cities in China.

**Cities**	**Total samples**	**Number of samples in month**					
		**1**	**2**	**3**	**4**	**5**	**6**
Beijing	30	1	9	4	8	5	3
Guangzhou	24	3	4	5	4	4	4
Chengdu	24	4	4	5	3	5	3
Weihai	16	2	2	4	3	2	3
Lanzhou	31	3	5	9	5	9	0
Jinhua	34	5	5	5	5	5	5
Zhengzhou	30	3	7	5	5	5	5
Harbin	18	3	3	3	3	3	3

### Protein Digestion

The procedure of protein digestion was described as previously (15). In brief, the HM samples were centrifuged at 3,000 rpm at 10°C for 30 min. The fat layer was removed and the remaining parts of protein concentration were measured using the BCA kit. Based on the results of the BCA, 10 μl proteins (1 μg/μl) was diluted with 100 μl 0.05 M ammonium bicarbonate in 0.5 ml Eppendorf tube. Approximately, 10 μl 0.1 M DTT was added. The mixture was kept in 56°C water-bath for 30 min, followed by adding 15 μl 0.5 M Iodoacetamide (IAA). The mixture was incubated at room temperature for 30 min in the dark. The protein digestion was performed by adding a mass ratio of 1:100 trypsin/protein and mildly shook at room temperature overnight. The digestion was stopped by adding 1% formic acid. Before running in LC-MS, the samples were desalted using C-18 column.

### Liquid Chromatography–Mass Spectrometry

The proteomics was performed using EASY-nLC 1200 coupled with Q Exactive HF. Generally, the samples were separated using a C18 analytical column (150 μm inner-diameter, outer-diameter 15 cm−1.9 μm, 120Å pore size, ReproSil-Pur C18-AQ). The mobile phase was constituted by 0.1% formic acid in water and 0.1% formic acid as solution A; and 19.9% water mixed with 80% acetonitrile as solution B. The flow rate was 0.6 μl/min, the column temperature was 50°C, the gradient was 4–7% solution B at the initial 1 min, 7–13% solution B for 1–7 min, 13–25% solution B for 7–47 min, 25–40% solution B for 47–68 min, 40-955 solution B for 68–69 min, and keeping 95% solution B for 69–75 min. The MS setting was 2.1 kV spray voltage. MS data were acquired by using data-dependent acquisition mode which dynamically chose the top-30 most abundant precursor ions from survey scan (300–1,400 *m/z*) for high energy collisional dissociation (HCD) fragmentation with a resolution of 120,000 (200 *m/z*). The MS/MS spectra were acquired in the HF normal scan mode.

### Data Analysis

The results from LC-MS/MS raw files were analyzed by using Maxquant 1.6.3.4 (9, 16). The *Homo sapiens* proteome database was downloaded from Uniprot (https://www.uniprot.org) and configured by Maxquant. In addition, the contamination database of Maxquant was adopted.

The carbamide-methylation of cysteine was set as fixed modification, and oxidation of methionine, N-terminal acetylation, and deamidation of asparagine or glutamine was set as variable modifications. Mass tolerance was set as 20 ppm for MS peaks and 0.5 Da for MS/MS peaks. The false discovery rates (FDR) was set as 1% and at least 1 peptide was required for identification. Label-free quantification (LFQ) and intensity-based absolute quantification (iBAQ) values were selected for relative protein quantification across all the samples and comparing the levels of different proteins from the same sample, respectively.

### Statistical Analysis

The significant differences were analyzed using SPSS 22 (IBM, USA), and the test used was one-way ANOVA with *post-hoc* Tukey's HSD (*P* < 0.05). Protein differentiation and principal component analysis (PCA) were performed by XLSTAT 2021 with the Benjamini-Hochberg test (*P* < 0.05).

### Cluster and Gene Ontology Enrichment Analysis

The GO enrichment of protein was performed by using DAVID Bioinformatics resources 6.8 (https://david.ncifcrf.gov) (9, 17). The protein–protein interaction was figured out by using STRING 9 (18).

## Results

### Identified Proteins in Eight Cities

In the present study, 860, 760, 1,426, 1,298, 1,029, 1,029, 1,022, and 960 proteins were identified in Beijing, Guangzhou, Chengdu, Weihai, Lanzhou, Jinhua, Zhengzhou, and Harbin, respectively. The list of identified proteins can be seen in Supplementary Table 1.

**Figure 1:**
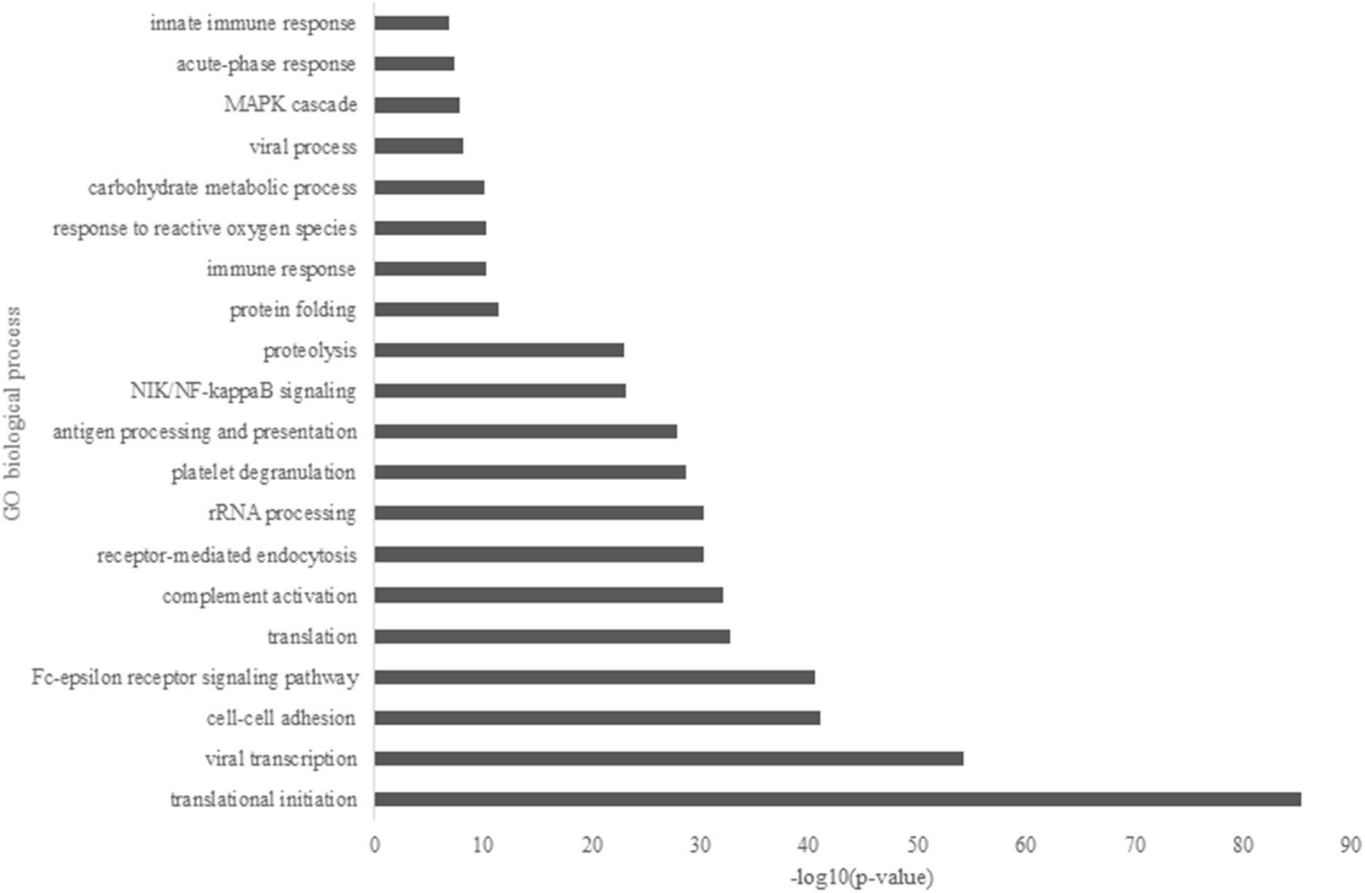
Major gene ontology (GO) biological process of overall identified proteins in Chinese human milk (HM) enriched by the DAVID bioinformatics.

Major GO biological processes from the overall identified protein (Figure 1) showed that translational initiation had the dominant function in HM proteomes with a *p*-value of 4.52 x 10^−86^. It was followed by viral transcription, cell-cell adhesion, Fc-epsilon signaling pathway, translation, complement activation, and receptor-mediated endocytosis. The metabolism function was represented by terms of proteolysis and carbohydrate metabolic process. Several immune related functions showed to be significant in the GOBP enrichment, such as antigen processing and presentation, immune response, and innate immune response with a *p*-value of 1.28 x 10^−28^, 4.42 x 10^−11^, and 1.40 x 10^−7^, respectively.

### Protein Variation Across the Cities and Lactation Periods

**Figure 2:**
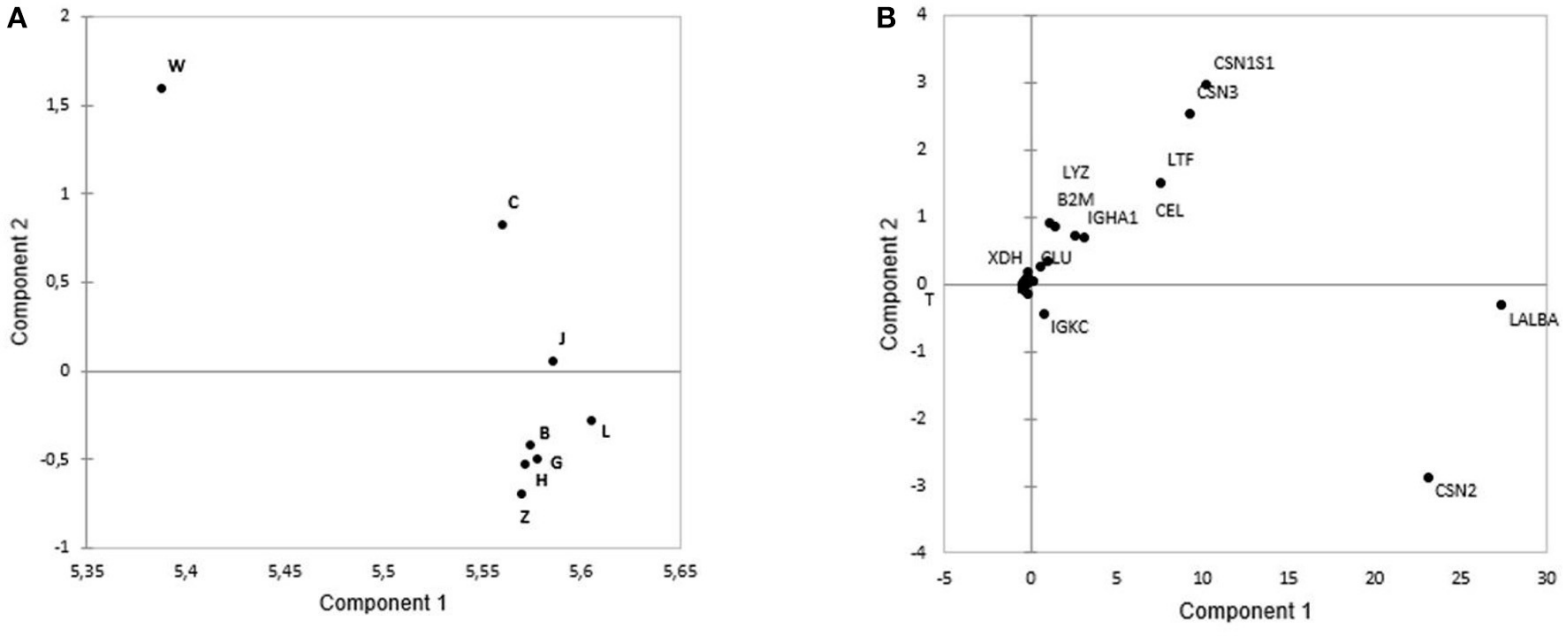
The principal component analysis (PCA) plots based on protein intensity of: **(A)** B, Beijing; G, Guangzhou; C, Chengdu;W, Weihai; L, Lanzhou; J, Jinhua; Z, Zhengzhou, and H, Harbin. Weihai, Chengdu, and Jinhua are located in the same pool, while Lanzhou, Beijing, Guangzhou, Harbin, and Zhengzhou are located in the second pool. **(B)** Loading plots based on the intensity of the proteins showed that CSN2, LALBA, and IGKC were the abundant proteins in the second pool, while the first pool had abundance of CSN1S1, CSN3, LTF, LYZ, B2M, IGHA1, CEL, CLU, B2M, and XDH.

The PCA showed from principal component 1 (PC1) and principal component 2 (PC2) could explain 99.37% of intensity-based protein variance among eight cities. From PC1 and PC2, the first pool consisted of Weihai, Chengdu, and Jinhua, which could be discriminated from the second pool that consisted of Zhengzhou, Beijing, Guangzhou, Lanzhou, and Harbin (Figure 2A). Those discrimination were based on several protein including LALBA, CSN2, and IGKC that were higher in the second pool. Meanwhile, CSNS1, CSN3, LTF, CEL, IGHA1, CLU, B2M, LYZ, and XDH were abundant in the first pool (Figure 2B).

**Figure 3:**
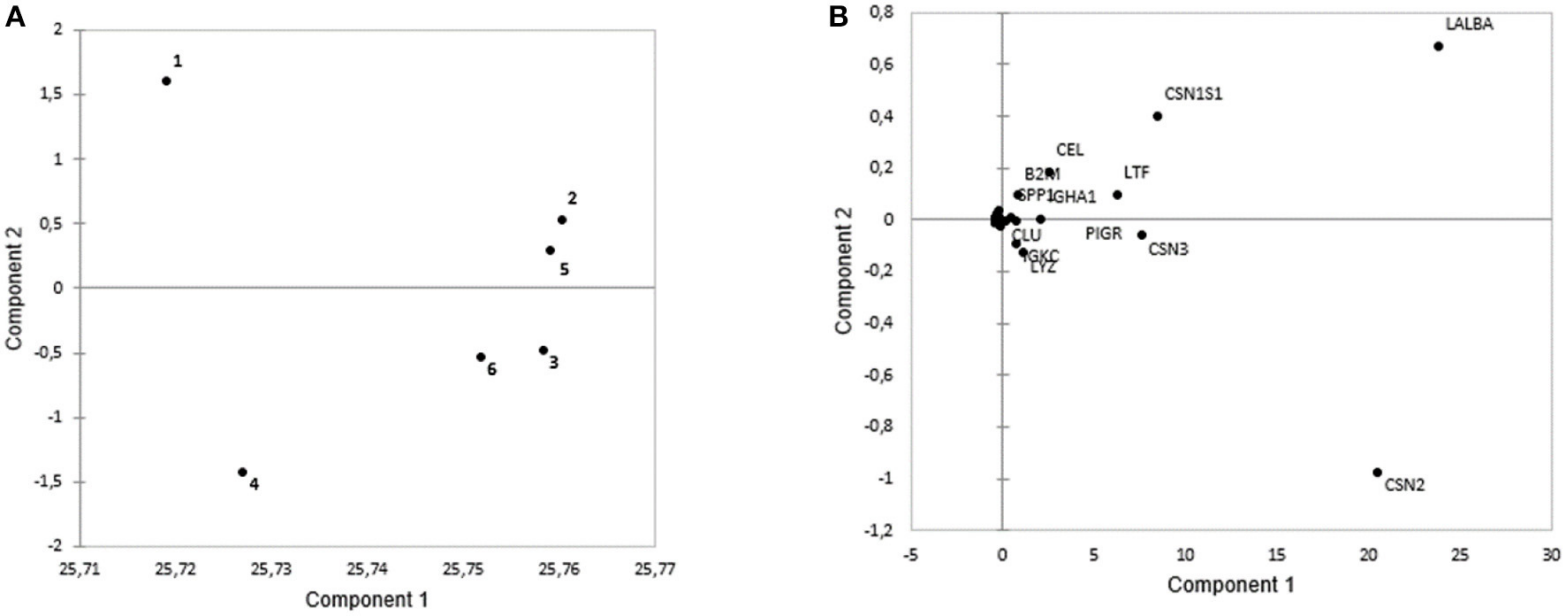
The PCA plots based on protein intensity of: **(A)** Different lactation periods during the 6 months of lactation. The first pool includes the 1st, 2nd, and 5th month of lactation, while second pool includes the 3rd, 6th, and 4th month of lactation. **(B)** Loading plots based on intensity of the proteins based on the different lactation periods. The first pool was abundant in LALBA, CSN1S1, CEL, LTF, B2M, SPP1, and IGHA1. Meanwhile, the second pool was abundant with CLU, PIGR, IGKC, LYZ, and CSN.

Proteins among different lactation periods were figured out by using PC1 and PC2 that could explain 99.95% of intensity-based protein variation (Figure 3). The first pool consisted of the 1st, 2nd, and 5th months of the lactation period, while the other groups consisted of the 3rd, 4th, and 5th months of the lactation period (Figure 3A). These groups were discriminated by several proteins, including LALBA, CSN1S1, CEL, LTF, B2M, SPP1, and IGHA1 that were abundant in the first pool. The second pool had a higher abundance of CSN2, CSN3, LYZ, IGKC, CLU, and PIGR (Figure 3B). The seven major proteins in all the cities were LALBA, CSN2, CSN1S1, CSN3, LTF, ALB, and CEL. The average intensity-based absolute quantification (iBAQ) value and the putative function of each protein based on the DAVID Bioinformatics were available in Table 2. Among the identified proteins, LALBA shared the highest percentage than the rest of the proteins. Zhengzhou had the highest LALBA concentration. LALBA and CSN2 in Weihai were below the other provinces on average, while the kappa-casein was the highest. Bile salt-stimulated lipase (CEL), as the seventh major protein in HM across the provinces, was noticed as the highest in Jinhua.

**Table 2:** Most abundant protein identified in eight cities.

**Gene names**	**Average iBAQ (%)** ^**a**^ **across the cities** ^**b**^								**Putative function**
	**B**	**G**	**C**	**W**	**L**	**J**	**Z**	**H**	
LALBAAlpha-lactalbumin	23.61 ± 3.36	24.71 ± 3.82	21.43 ± 3.81	18.07 ± 4.90	25.11 ± 4.59	27.73 ± 4.71	30.20 ± 2.86	29.68 ± 2.22	Enzyme: apoptotic process, cell-cell signaling, defense response to bacterium, lactose biosynthetic process, signal transduction
CSN2Beta casein	25.83 ± 6.63	26.75 ± 6.20	15.65 ± 6.56	12.27 ± 4.20	22.39 ± 8.04	19.08 ± 5.58	26.69 ± 4.66	22.70 ± 2.64	Transport: calcium ion transport, lactation, negative regulation of-cystein type endopeptidase
CSN1S1Alpha casein S1	10.41 ± 1.90	8.71 ± 2.73	10.96 ± 1.93	12.02 ± 2.70	8.42 ± 1.82	8.37 ± 1.56	8.66 ± 1.35	7.25 ± 1.82	Transport: response to dehydroepiandrosterone, response to estradiol, response to progesterone, transmembrane transport
CSN3Kappa casein	7.80 ± 1.21	8.67 ± 1.35	9.51 ± 2.50	10.50 ± 3.12	7.41 ± 1.50	9.36 ± 1.55	6.94 ± 1.09	7.81 ± 1.29	Transport: lactation, protein stabilization, transmembrane transport
LTFLactotransferrin	6.40 ± 1.35	6.76 ± 1.45	7.22 ± 1.15	7.91 ± 1.35	7.52 ± 1.64	7.14 ± 1.41	6.66 ± 1.03	6.75 ± 0.86	Immune: antibacterial humoral response, innate immune response, cellular protein metabolic process, retina homeostasis
ALBAlbumin	4.32 ± 0.82	5.05 ± 0.80	6.92 ± 1.19	7.32 ± 1.64	3.35 ± 1.28	3.88 ± 1.13	4.73 ± 0.72	5.23 ± 0.60	Transport: platelet degranulation, cellular protein metabolic process, receptor-mediated endocytosis
CELCarboxyl-ester-lipase	3.36 ± 1.17	2.90 ± 0.78	3.44 ± 1.27	3.28 ± 0.92	3.35 ± 1.27	3.88 ± 1.13	2.08 ± 0.66	2.61 ± 2.90	Enzyme: fatty acid catabolic process, chemical synaptic transmission, lipid digestion, lipid metabolic process

a *Accounted as average of iBAQ during 6 months of lactation*.

b *B, Beijing; G, Guangzhou; C, Chengdu; W, Weihai; L, Lanzhou; J, Jinhua; Z, Zhengzhou; H, Harbin*.

The intensity of 184 proteins was significantly different among the cities (Supplementary Table 2), including several abundant proteins: CSN1S1, CSN2, CSN3, LTF, CEL, IGKC, IGHA1, B2M, and PIGR. Four proteins were significantly different during 6 months of lactation, namely CTSC, LYZ, CA6, and CHRDL2 (Supplementary Table 3). As the lactation prolonged, the intensity of CTSC, CA6, and CHRDL2 decreased, while the intensity of LYZ increased.

### Gene Ontology Enrichment of Significantly Different Proteins

**Figure 4:**
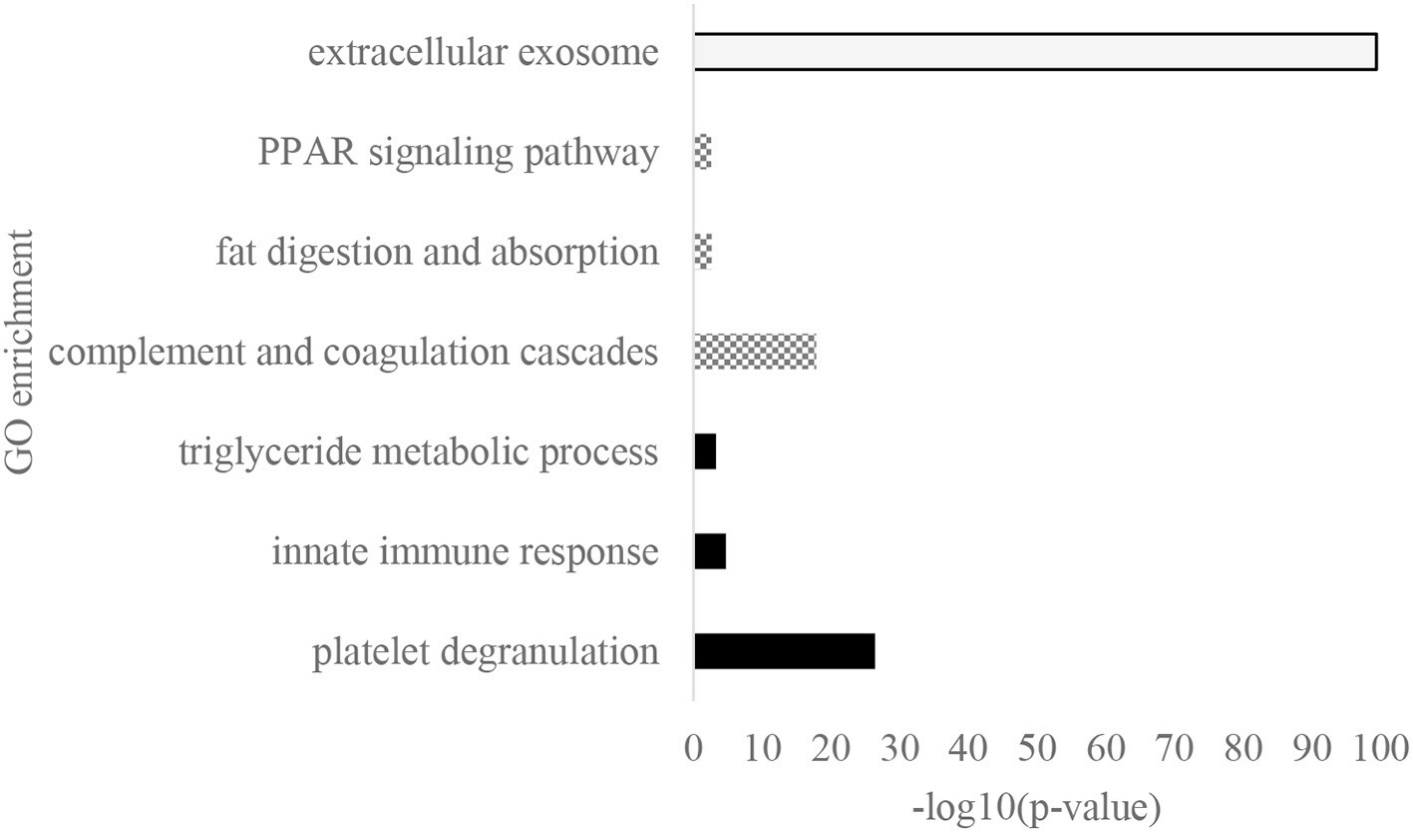
The GO enrichment of significantly different protein across eight cities. White: GO cellular location, dominated by extracellular exosome; Gray: Kyoto Encyclopedia of Genes and Genomes (KEGG) pathways that consisted of complement coagulation cascades, fat digestion and absorption, and Peroxisome proliferator-activated receptors (PPAR) signaling pathway; and Black: GO biological process, dominated by platelet degranulation, innate immune response, and triglyceride metabolic process.

Gene ontology biological process (GOBP), cellular (GOCC), and Kyoto Encyclopedia of Genes and Genomes (KEGG) pathways were enriched from the significantly different proteins across the cities (Figure 4). Platelet degranulation was the most noticeable GOBP observed that had a *p*-value of 3.66 x 10^−27^, and it was followed by innate immune response and triglyceride metabolic process with a *p*-value of 1.87 x 10^−5^ and 5.38 x 10^−4^, respectively. The white bar showed the extracellular exosome as the major GOCC, and the KEGG pathway was dominated by complement and coagulation cascades, fat digestion and absorption, and peroxisome proliferator-activated receptors (PPAR) signaling pathway.

**Figure 5:**
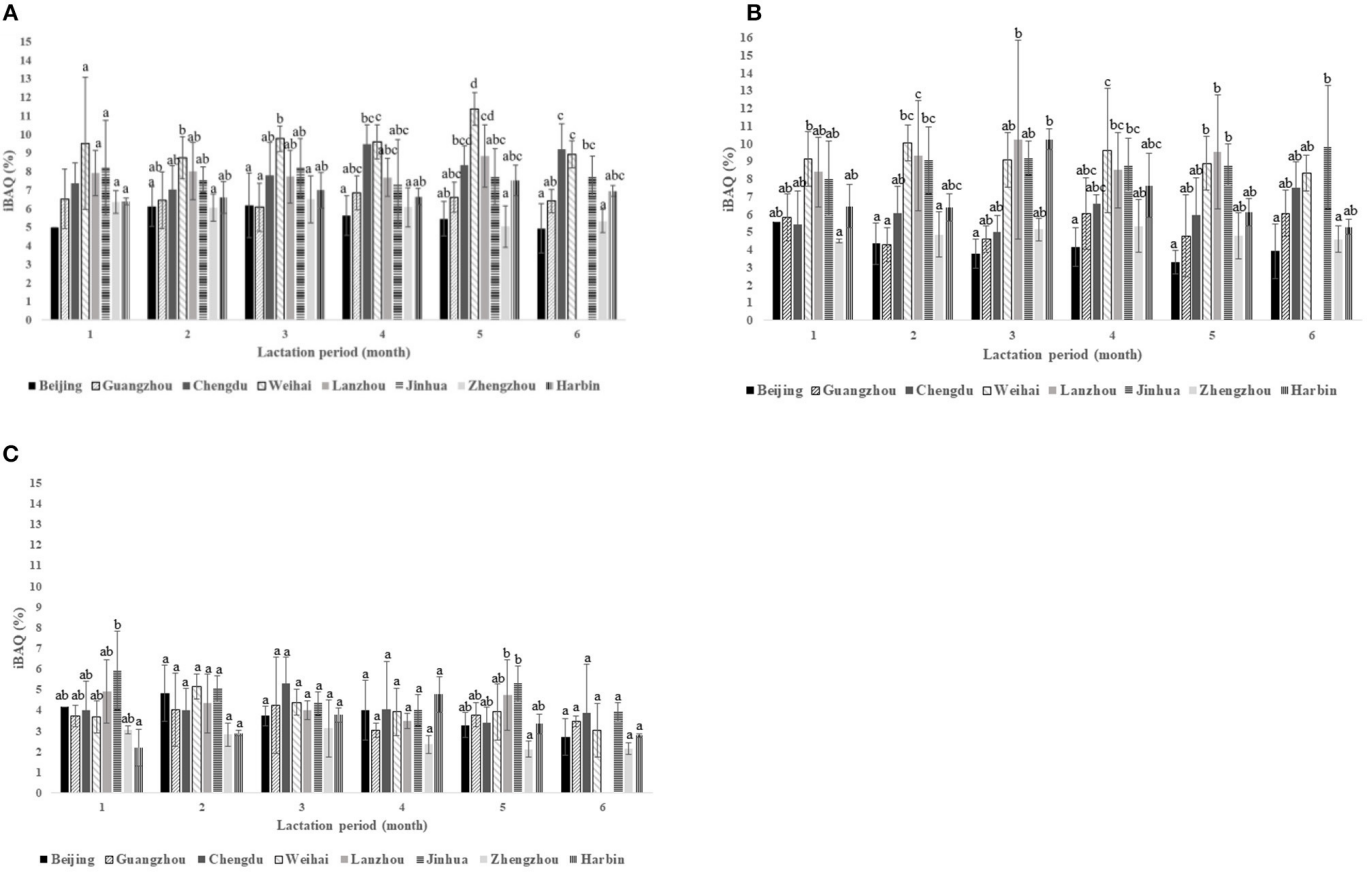
The summed intensities of GO biological process in the terms of: **(A)** platelet degranulation; **(B)** innate immune response, and **(C)** triglyceride metabolic process periods showed the most varied intensities values among the cities.

The intensity of proteins enriched in platelet degranulation, triglyceride metabolic process, and innate immune response is summed in Figure 5. Figure 5A shows the obvious different intensities in platelet degranulation across eight cities except in the 1st month of lactation which ranged from 5 to 11% of the iBAQ values. Weihai had the highest iBAQ values in the term of platelet degranulation during the 1st to 5th months of the lactation period. Meanwhile, in the 6th month Weihai and Chengdu were not significantly different based on the HSD *post-hoc* test. Zhengzhou consistently had the lowest intensities during the periods, while Harbin also shared the insignificant difference with Zhengzhou except in the 5th month of lactation.

Remarkable different protein intensities were found during 6 months of the lactation period in terms of innate immune response that ranged from 4 to 11% of the iBAQ values (Figure 5B), especially in Weihai, Lanzhou, and Jinhua, which had higher intensities among the other cities. In contrast, Beijing and Zhengzhou were lower in iBAQ values compared with the others. In this term, the significant variation was observed along the lactation period in each city. The 2nd and 4th months had more variation intensities compared to that of the other periods.

The triglyceride metabolic process, which ranged from 2 to 6% of the iBAQ values, revealed a significant difference in the 1st and 5th months of the lactation period (Figure 5C). Meanwhile, no significant difference was observed during the rest of the lactation period. Harbin had the lowest intensities in the 1st month of the lactation period; in contrast, Jinhua was the highest. However, in the 5th month, Zhengzhou had the lowest iBAQ values, and Jinhua still had the highest.

**Figure 6:**
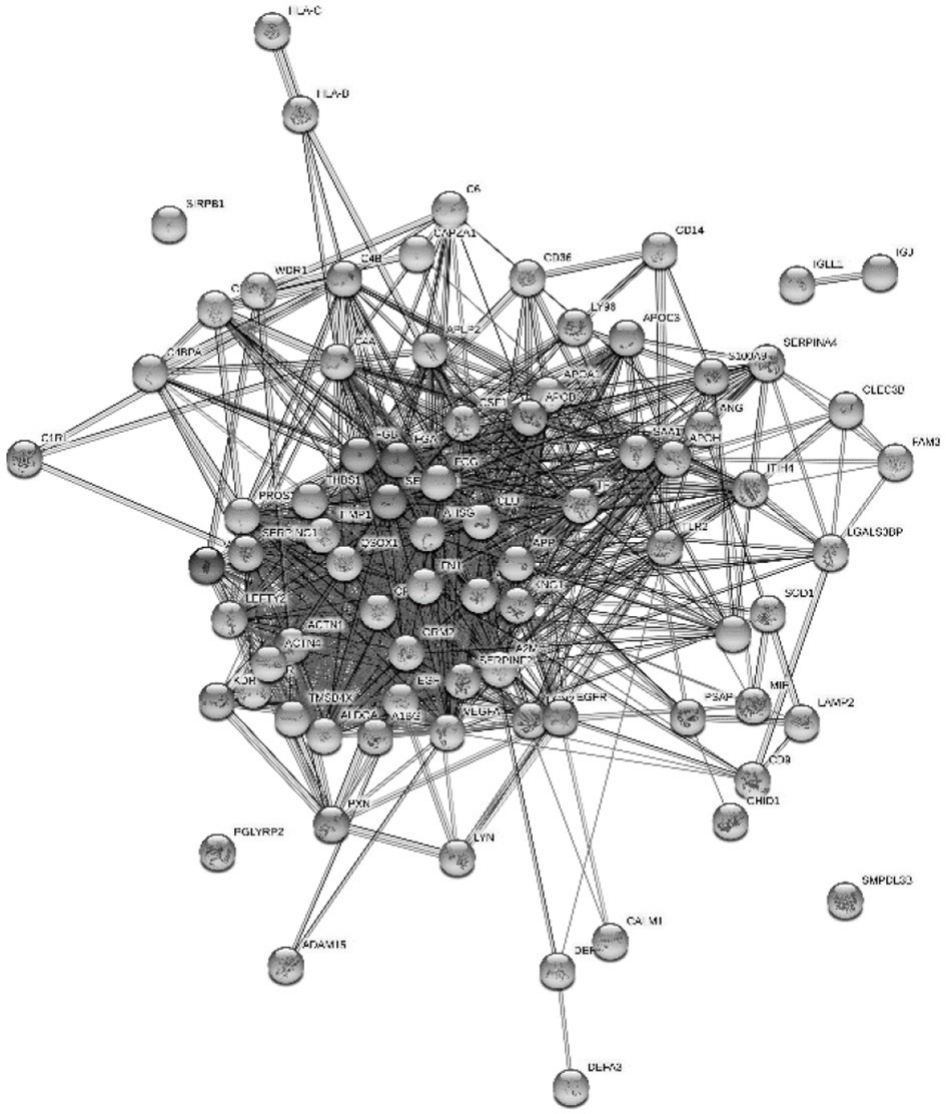
Interaction of platelet degranulation and innate immune response protein in Chinese HM were enriched with DAVID Bioinformatics and visualized using STRING 9.

The proteins from platelet degranulation and innate immune response were illustrated by STRING 9 to see the protein–protein interactions since both the terms were enriched under the complement and coagulation cascade in the KEGG pathways (Figure 6). Almost all of the proteins were connected except the SMPDL28, SIRPB1, IGLL, and IGJ.

## Discussions

### Human Milk Proteome Varied Across the Cities and Lactation Periods

Human milk composition is suited for infant needs and growth. However, the profile of HM is crucial for developing products for infants who either cannot access their mother's milk or whose mothers' milk production volume was insufficient. The HM variation in the different continents had been reported previously using the metabolomics method (11). The present method found a higher number of identified proteins than the previous study (13). It means our proteomics method was comparable to the other methods and could be deeper in the bioinformatics enrichment since more proteins were identified to interpret its biological function.

Twelve proteins were the discriminants of the cities, namely LALBA, CSN2, IGKC, CSN1S1, CSN3, LTF, CEL, IGHA1, B2M, LYZ, CLU, and XDH. These discriminant proteins were mainly contributed as host defense in the biological process of an infant. It was agreed with a previous research that found the immune protein including lactalbumin, lactoferrin, and IgA were varied among the population as a function of pathogen pressure of the environment (19).

In the most abundant protein in HM, this study found that CSN1S1, CSN2, and CSN3, which belonged to casein, were statistically different across the cities. Casein plays the role of primary source of phosphate and calcium in HM due to its function in casein-micelle aggregates in the calcium transport process (20, 21). This finding was in line with a prior study that reported the remarkable difference of kappa and beta casein in HM serum of Chinese and Dutch mothers (14). The LTF variations across the cities were in line with previous findings in Chinese HM across distinct regions (22).

Based on differential expression tests, variation of significant proteins across the cities was very high that presented in Supplementary Table 2. A total of 184 proteins were significantly different across the city with a *p*-value of below 0.05 that was followed by enrichment in Figure 4. Meanwhile, only four proteins were statistically different during the 6 months of lactation, namely CTSC, LYZ, CA6, and CHRDL2. LYZ was upregulated while CTSC, CA6, and CHRDL2 were downregulated. The downregulated CHRDL2 was in line with a prior proteomics research in HM over lactation (3). Since the function of CHRDL2 is related to ossification, it might be possible that the expression was downregulated by organ maturation throughout the 6 months. The increase of LYZ in the human mature milk was previously described by Montagne et al. as the passive protective agents of breast-fed infants during mature lactation (23).

In Figure 2, the first pool consisted of the 1st, 2nd, and 5th months of the lactation period that had a high abundance of LALBA, CSN1S1, CEL, LTF, B2M, SPP1, and IGHA1. The high abundance of LALBA and CSN1S1 in the beginning months of the lactation had been previously reported in bovine milk (24). B2M, IGHA1, and LTF as the proteins related to antibacterial humoral response were logical to have a high abundance in the 1st and 2nd months of lactation since newborns had little protection against bacteria from the environment. CEL, as the main protein related to fat metabolism, was reported to be highly abundant during the initial months (3) since it functions as an immature pancreatic lipase substitution. Meanwhile, the highly percentage of aforementioned proteins in the fifth month needs to be further investigated. We speculated it was because of the highly individual variation in the 5th month samples.

### Significant Different Proteins Across the Cities Were Linked to Newborn Protection

The significantly different proteins across the cities were involved in platelet degranulation, and those proteins were mainly located in the extracellular exosome (Figure 4). It was agreed with a previous finding in proteomics of bovine milk exosome that showed platelet degranulation was the most significant biological process (25). Beyond its primary role in thrombus or plug formation in wound healing, the platelet also worked in tissue repair, angiogenesis, inflammation, and host defense (26–30). In newborns, overall platelet degranulation released during platelet activation was lower compared to adults (31). Based on our results, there was no significant difference in platelet degranulation in the 1st month across the cities (Figure 5A). It might indicate that the newborns demanded HM proteins to regulate platelet degranulation for completing platelet function since platelets activation was low in the first few days of life (32).

Neonates were generally believed to have partially immunological incompetence and were susceptible to infections. Platelets also played a role in the host defense of the newborns. By the present result, platelet degranulation shared similar proteins with the innate immune response in GOBP, including FGA, FGB, CLU, SERPING1, and CD36. All proteins contributed in both terms were figured out in protein network connection (Figure 6). Platelet glycoprotein 4 (CD36) had a responsibility in the phagocytosis process (31). Phagocytosis was an important innate immune response process to protect infants from microbes. CLU is a highly glycosylated protein that was linked to cell damage and apoptosis. This protein was found to be overexpressed at stressed tissues to provide a chaperone-like activity and prevent other proteins from damage (33). FGA is known as the main protein in handling fibrin production as one of the primary components of blood clots. It also acts as a fibrin deposition process that is associated with infection, where it protects against IFNG-mediated hemorrhage. The link of platelet degranulation and immune response as the host defense were regulated in complement and coagulation cascades, which were enriched in major KEGG pathway (Figure 4).

Immune response plays important roles in infants, especially innate immune response in newborns since adaptive immune response is not well-developed yet. The function of HM as a complementary immune system for newborns has been previously reported (34). From our identified protein (Supplementary Table 1), above 54 proteins were enriched as innate immune response in biological process with *p*-value 1.6 x 10^−10^. It was higher than previous findings that used a similar GO enrichment tool in HM (8). That research found the *p*-value of the immune system to be 1.8 x 10^−7.^ The reason might be because this study had higher identified proteins. Based on the significant expressed protein enrichment, the *p*-value of the innate immune response was 1.87 x 10^−5^,which consisted of 17 proteins, namely FGB, FGA, IGHM, CFI, CLU, IGHG4, C4A, IGHG1, IGHG2, IGKC, IGLL1, SERPING1, CD14, IGHA1, SMPDL3B, IGHA2, and B2M. These 17 proteins brought variation to the quantitative amount of innate immune response across the cities (Figure 5B) that showed significant variations in each period of the lactation. The differences of immune-related protein intensity were also previously found in the different geographic location and ethnicity in China because the environment could influence the pathogen (13). However, other factors such as the health condition or infection of the infant should be further observed, since the infant infection could upregulated immune proteins in the HM, especially in the first year of lactation (35, 36).

### Triglyceride Metabolism Variations Across the Cities

Breastfeed infants have better lipid utilization compared to formula-feed infants (37–39). Since delivery, infants used fat as the major energy source that contributed about 40–55% of the total energy and could obtain 5.5 kg fat intake during 6 months of lactation (40, 41). Triglycerides represented 98–99% of total fat in HM (42). Fat metabolism and fat absorption were noticed as the enriched KEEG pathway from the significantly expressed proteins (Figure 4). The crucial protein that handled fat metabolism in HM was CEL, which was found to be the seventh abundant protein (Table 2) and also significantly expressed protein across the cities (Supplementary Table 2). CEL from HM played a major role in infant lipid utilization by replacing pancreatic triglycerides lipase (PTL), which is secreted by the pancreas, while the pancreas condition is immature (38, 43). The present research found five significantly expressed proteins in different cities, namely CEL, APOH, APOA2, LPL, and APOE which were enriched in the triglycerides metabolic process. These proteins brought remarkable variation in the triglyceride metabolic process (Figure 5C), particularly in the 1st and 5th months of the lactation period. This could be noteworthy because the lower abundance of the triglyceride metabolic process proteins might lead to lower weight gain of the infant since fat should be the major energy intake. In addition, the immature digestion tract of the infant needs HM protein to aid fat digestion (43).

However, bigger samples in a longitudinal study during the lactation stages will give more benefit to fill the gaps in this research as well as decrease the variations among individual mothers. It will bring a deeper quality of biological role interpretation as the basic data for developing the most suitable infant formula for babies who cannot obtain HM in the exclusive breastfeeding period.

## Conclusion

This present research found a noticeable variation of HM proteins across eight cities in China and four significantly expressed proteins during the 6 months of lactation. The 184 significantly expressed proteins across the cities mainly influenced the infant biological process in terms of platelet degranulation, innate immune response, and triglyceride metabolic process. This research could be a good recommendation for developing specialized region infant formula or HM fortifiers during the exclusive breastfeeding period.

## Data Availability Statement

The raw data supporting the conclusions of this article will be made available by the authors, without undue reservation.

## Ethics Statement

The studies involving human participants were reviewed and approved by National Library of Medicine. The patients/participants provided their written informed consent to participate in this study.

## Author Contributions

JLu: conceptualization and validation. WZ and JLu: methodology. RS and WZ: software and investigation. WZ: formal analysis. JLu, JP, and YL: resources. RS: data curation, writing—original draft preparation, and visualization. HZ, XP, SZ, and JLu: writing—review and editing. JLu and JLv: supervision. SJ, JLu, and JLv: project administration. JP, SJ, JLu, and JLv: funding acquisition. All authors have read and agreed to the published version of the manuscript.

## Conflict of Interest

JP, YL, and SJ were employed by the company Heilongjiang Feihe Dairy Co., Ltd. The remaining authors declare that the research was conducted in the absence of any commercial or financial relationships that could be construed as a potential conflict of interest.

## Publisher's Note

All claims expressed in this article are solely those of the authors and do not necessarily represent those of their affiliated organizations, or those of the publisher, the editors and the reviewers. Any product that may be evaluated in this article, or claim that may be made by its manufacturer, is not guaranteed or endorsed by the publisher.
